# Virtual Reality as a Stress Measurement Platform: Real-Time Behavioral Analysis with Minimal Hardware

**DOI:** 10.3390/s25175323

**Published:** 2025-08-27

**Authors:** Audrey Rah, Yuhua Chen

**Affiliations:** Department of Electrical and Computer Engineering, University of Houston, Houston, TX 77204, USA; yuhuachen@uh.edu

**Keywords:** virtual reality, stress detection, behavioral analysis, physiological sensors, GSR, low-cost sensors

## Abstract

With the growing use of digital technologies and interactive games, there is rising interest in how people respond to challenges, stress, and decision-making in virtual environments. Studying human behavior in such settings helps to improve design, training, and user experience. Instead of relying on complex devices, Virtual Reality (VR) creates new ways to observe and understand these responses in a simple and engaging format. This study introduces a lightweight method for monitoring stress levels that uses VR as the primary sensing platform. Detection relies on behavioral signals from VR. A minimal sensor such as Galvanic Skin Response (GSR), which measures skin conductance as a sign of physiological body response, supports the Sensor-Assisted Unity Architecture. The proposed Sensor-Assisted Unity Architecture focuses on analyzing the user’s behavior inside the virtual environment along with physical sensory measurements. Most existing systems rely on physiological wearables, which add both cost and complexity. The Sensor-Assisted Unity Architecture shifts the focus to behavioral analysis in VR supplemented by minimal physiological input. Behavioral cues captured within the VR environment are analyzed in real time by an embedded processor, which then triggers simple physical feedback. Results show that combining VR behavioral data with a minimal sensor can improve detection in cases where behavioral or physiological signals alone may be insufficient. While this study does not quantitatively compare the Sensor-Assisted Unity Architecture to multi-sensor setups, it highlights VR as the main platform, with sensor input offering targeted enhancements without significantly increasing system complexity.

## 1. Introduction

Real-time stress detection can enhance training and safety in high-pressure environments by providing timely feedback and performance monitoring [1,2,3].

Two primary modalities are commonly used: behavioral and physiological data. Behavioral signals arise from a user’s interactions within Virtual Reality (VR) environments and may manifest as hesitation, trembling, or repeated task failure [4,5]. By contrast, physiological signals are captured with wearable sensors such as skin-conductance electrodes or heart rate monitors that track autonomic changes in sweat activity or cardiovascular functions. Previous studies have demonstrated that autonomic arousal can be inferred from skin conductance alone, supporting closed-loop applications without requiring complex sensor arrays [6,7]. Physiological data such as heart rate and sweating can provide helpful information, but may not always be clear, as changes may also result from excitement, caffeine intake, exercise, or hot weather [8,9].

In VR, the environment is fully programmable, allowing researchers to introduce well-defined high-stress conditions such as flashing alarms, countdown timers, and sensory overload with precise timing. Because the exact moment a stressor is introduced is known, behavioral responses can be interpreted with confidence [9]. If a user hesitates, commits repeated errors, or shows physical signs such as hand tremors immediately after a stressor, these become strong behavioral indicators of cognitive load. The behavioral response is tied to a controlled VR trigger. When physiological sensors are used alongside VR, their readings gain contextual meaning. If skin conductance and heart rate signals rise as behavioral markers appear during a known stressor, then it is possible to confidently conclude that a real stress response occurred [7]. Conversely, when behavioral signs are weak or absent but internal physiological changes are detected by sensors, this can reveal hidden stress that might otherwise be missed.

No prior system has combined precise VR-triggered behavioral monitoring with on-demand physiological sensing in a single low-latency framework. Therefore, in this paper, we propose the design of a Sensor-Assisted Unity Architecture that uses standard VR headsets and controllers to monitor natural reactions such as task failure, delay, or non-responsiveness. Our proposed Sensor-Assisted Unity Architecture incorporates a minimal, low cost wearable sensor to improve accuracy. This architecture maintains system simplicity while enhancing precision. A combined approach is particularly well-suited for environments that demand rapid feedback, precise tracking, and scalable tools. A system that mostly uses VR behavior with few sensors provides a useful and reliable way to measure stress. The main contributions of this paper are as follows:

- A Sensor-Assisted Unity Architecture for real-time stress detection without bulky wearables.
- A decision-level Sensor-Assisted Unity Architecture algorithm that invokes a single low-cost Galvanic Skin Response (GSR) sensor.
- An end-to-end pipeline achieving sub-120 ms latency.

The structure of this paper is as follows: Section 1 introduces the motivation and objectives of the study; Section 2 reviews the research background on physiological and behavioral sensing in VR, including limitations of wearable-based systems and advances in lightweight behavioral modeling; Section 3 presents the proposed Sensor-Assisted Unity Architecture, outlining its main components and data flow; Section 4 describes the implementation, covering behavioral feature extraction, logic, and real-time feedback mechanisms; Section 5 presents the experimental setup and results, including detection accuracy, latency measurements, and architectural comparisons across various stress scenarios; finally, Section 6 reflects on the findings and offers concluding remarks.

While the primary focus of this study is behavioral analysis in VR, we acknowledge the importance of validating stress states against established references. In this work, stress conditions were validated through synchronization with controlled VR triggers and confirmed by GSR fluctuations. To support future extensions, we suggest incorporating validated psychological scales such as the State–Trait Anxiety Inventory (STAI) [10] or the Perceived Stress Scale (PSS) [11] along with physiological baselines including Heart Rate Variability (HRV) [12] and cortisol sampling [13] as independent ground truths for more rigorous confirmation of stress. This limitation is further discussed in Section 5. In addition, the Sensor-Assisted Unity Architecture was evaluated using the Wearable Stress and Affect Detection (WESAD) dataset, demonstrating its ability to generalize beyond VR-generated data. However, the primary validation in this study remains based on controlled VR triggers, with stress confirmed through GSR fluctuations. The module used was the Grove GSR sensor (Model 101020052; Seed Studio, Shenzhen, China).

## 2. Research Background

### 2.1. Physiological Sensing in Virtual Environments

VR is widely adopted for simulation and high-risk training tasks. Accurate monitoring of cognitive stress and mental workload is essential in these scenarios. Existing VR monitoring systems often rely on physiological signals such as Electroencephalography (EEG), heart rate (HR), and oxygen saturation, which show strong correlations with self-reported stress levels [14,15]. Wickramasuriya et al. [7] introduced a deconvolution-based method to extract neural impulses from skin conductance signals, enabling real-time sympathetic arousal estimation in closed-loop feedback systems. Their results showed that changes in skin conductivity due to perspiration are reliable and low-cost indicators of stress and fatigue during cognitive tasks. Using standard VR hardware such as headsets and hand controllers, it is possible to capture behavioral signals such as reaction time, mistakes, and unusual movements without requiring invasive body sensors [15,16]. VR environments can reproduce high-stakes tasks, time pressure, and sensory overloads that induce measurable cognitive and emotional responses in users [17], making VR a useful tool for examining stress responses in scenarios that are difficult or unsafe to reproduce in real life.

### 2.2. Limitations of Wearable-Based Systems

Despite their usefulness, wearable devices can introduce discomfort and may interfere with natural motion. They often require frequent calibration, are sensitive to placement errors, and may limit user immersion during training. These limitations have motivated researchers to explore alternatives that are less invasive and more adaptable. Wearable sensors such as EEG caps and HR monitors have been extensively used for stress detection in both laboratory and field settings [16,18]. While these sensors provide valuable physiological data, their integration in VR environments often introduces practical challenges; for example, EEG systems can be sensitive to electrical noise from VR headsets, and motion artifacts can degrade signal quality during active tasks [17]. Signal accuracy and maintaining proper contact can also be considerations in dynamic VR tasks [19]. Several studies have highlighted the tradeoffs between sensor accuracy and user comfort in immersive environments. It is reported that EEG-based systems often require users to remain still for optimal data collection, which may not be feasible in dynamic VR training scenarios [14]. Physiological signals can be influenced by external factors such as temperature, humidity, and user-specific characteristics such as skin type or hydration levels [18]. These factors introduce variability and complicate data interpretation; moreover, wearable sensors can increase the complexity and cost of VR training systems. Maintaining proper sensor calibration, ensuring reliable data transmission, and preventing hardware interference can also impose additional burdens on system designers and users [16,17]. In time-sensitive or high-pressure training scenarios such as emergency response simulations, these issues can limit the feasibility of physiological sensing approaches. Therefore, there is growing interest in alternative methods that rely on behavioral signals and embedded system integration to provide practical, scalable, and user-friendly stress detection solutions in VR environments [15].

### 2.3. Shift Towards Behavioral Indicators

Behavioral signals such as reaction time, task completion accuracy, and motion irregularities have emerged as viable alternatives. These indicators can be passively captured using standard VR headsets and controllers. Unlike physiological sensors, behavioral data acquisition does not require specialized hardware. This enables lightweight, scalable, and cost-effective systems suitable for long-duration sessions. Recent research has demonstrated the value of behavioral signals as indirect but reliable proxies for cognitive load and stress. Task repetition, hesitation, and delays in VR simulations can accurately predict user stress levels, aligning well with self-reported data [16]. Motion irregularities, such as hand tremors and erratic movements are common in high-stress scenarios. These can be systematically analyzed for real-time feedback [20]. Integrating behavioral metrics into VR applications is feasible as well. These data streams can be captured continuously without interfering with immersion or comfort [15,17]. Moreover, behavioral indicators can provide a holistic understanding of user states by combining multiple metrics, such as response latency, error frequency, and path deviations [18]. This multifaceted approach enables the development of robust and adaptive feedback systems that adjust task difficulty or trigger warnings based on detected stress patterns [14,16]. Recent studies in stress detection have relied heavily on physiological measurements such as heart rate variability, electrodermal activity, and EEG signals. While these methods offer high-resolution insight into internal states, they often require complex, costly, or intrusive equipment. Although physiological signals offer granular insights into biological processes, behavioral indicators present a practical solution for many training scenarios, especially when ease of use, cost, and long-term wearability are critical factors [15]. Therefore, the use of behavioral data has become an attractive modality for stress monitoring in VR, supporting the design of scalable systems that promote user engagement while minimizing hardware complexity.

### 2.4. Enhancing Behavioral Models with Machine Learning

Recent advancements have integrated behavioral analysis with Machine Learning (ML) techniques to improve stress detection accuracy in virtual environments. ML models such as Convolutional Neural Networks (CNNs), Recurrent Neural Networks (RNNs), and decision trees have shown strong performance in identifying patterns of cognitive load, stress, and fatigue [15,17]. CNNs have been successfully applied to classify stress levels based on motion trajectories and task errors in VR simulations, achieving over 85% accuracy [17]. One key advantage of ML-based models is their ability to adapt across users, identifying subtle differences in behavior that traditional threshold-based systems may overlook. These advancements support the development of scalable, user-centered VR training systems capable of real-time adaptation and continuous learning based on behavioral feedback [14,19]. As ML models continue to evolve, their integration into VR-based behavioral monitoring systems holds significant promise for improving safety, engagement, and personalized user experiences in high-stakes training environments.

### 2.5. Ethical Considerations and Data Privacy

The increasing use of behavioral monitoring in VR and haptic systems introduces unique challenges for protecting sensitive user information [16,17]. Behavioral signals such as reaction patterns, hand tremors, and task errors can reveal intimate details about a user’s cognitive state, emotional responses, and even medical conditions [15,18]. Ethical frameworks emphasize the importance of informed consent, transparency in data usage, and clear communication of risks and benefits to users [19]. To address these concerns, researchers are exploring decentralized data processing methods that enable real-time analysis without transmitting raw sensor data to external servers. Techniques such as on-device processing, federated learning, and differential privacy can reduce the risk of data breaches while maintaining system functionality [14,17]. Zhang et al. demonstrated a VR stress detection system that processed behavioral signals locally on an Arduino microcontroller, eliminating the need for cloud-based storage [17]. In addition to technical solutions, ethical design principles encourage the minimization of data collection, secure storage practices, and anonymization techniques to protect user identities [16,18]. As VR systems become more immersive and data-rich, incorporating robust privacy safeguards will be essential for maintaining user trust and enabling widespread adoption across clinical, educational, and industrial settings.

### 2.6. Broader Applications and Haptic Interfaces

Behavior-driven monitoring systems have been applied beyond gaming to areas such as rehabilitation, surgical training, and robotics control [21]. Haptic feedback plays a critical role in these systems by enhancing realism and reinforcing behavioral cues [16,17]. For example, vibration patterns or resistive forces can support motor learning, procedural accuracy, and situational awareness in high-pressure scenarios [15]. Haptic interfaces also enable adaptive feedback control such as increasing vibration intensity when stress is detected or adjusting resistance based on user fatigue [14]. This multimodal approach enhances user engagement, safety, and performance across diverse VR applications, including medical simulations, emergency training, and collaborative robotics [17]. As VR technologies advance, integrating behavior-driven monitoring with haptic feedback holds significant potential for creating immersive, responsive, and user-centered training systems.

### 2.7. Towards Sensor-Assisted Unity Architectures and Minimal Systems

Current trends favor architectures that combine behavioral cues with simple physical sensors such as sweat patches. These systems balance detection accuracy and system complexity while improving usability and cost-effectiveness. This shift supports the broader goal of developing lightweight and user-friendly stress monitoring tools suitable for diverse VR applications. The proposed Sensor-Assisted Unity Architecture integrates basic sensors such as GSR, electrodes or temperature sensors with VR-based behavioral analysis to enhance detection accuracy while keeping system overhead low [16,18]. Kirschbaum and Hellhammer have demonstrated that combining skin conductance data with task error metrics improves sensitivity to stress variations compared to using either method alone. Minimal GSR integration has also been shown to enable adaptive feedback systems that adjust VR task difficulty based on real-time user states [19]. Sensor-Assisted Unity Architectures offer a practical way to develop scalable stress detection frameworks that work effectively. Such architectures can adapt to a variety of VR scenarios without requiring complex hardware or advanced technical expertise [14,18]. In contrast to existing systems, our work introduces a VR-centered framework in which behavioral cues serve as the primary input. Minimal sensor data are used only when necessary, making our Sensor-Assisted Unity Architecture lighter, more scalable, and better suited for real-time stress detection with minimal hardware. By using VR as the main platform for behavioral analysis and relying on sensors, these two sources of information are combined in a complementary and efficient way. This setup improves the system’s accuracy, reliability, and overall performance.

## 3. System Architecture

### 3.1. System Overview

This section introduces the design of our real-time stress detection called the Sensor-Assisted Unity Architecture. The system implements the Sensor-Assisted Unity Architecture algorithm, which integrates behavioral monitoring in a virtual reality environment with input from lightweight physiological sensors. The goal is to maintain a clear and consistent structure without switching between different system setups.

**Figure 1:**
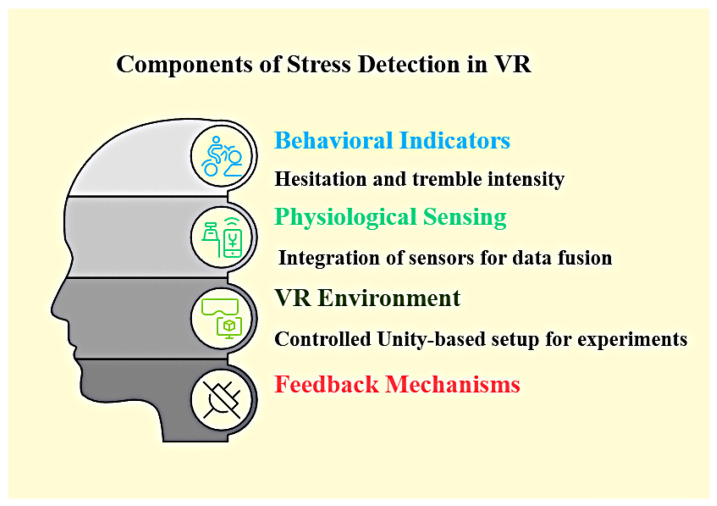
Key components of the VR-based stress detection system.

Figure 1 illustrates the key components of the proposed VR-based stress detection system. The system operates in a Unity-based VR environment, running the Sensor-Assisted Unity Architecture algorithm. Behavioral indicators, such as hesitation and tremor intensity, are always monitored as users interact with the virtual world. In addition, physiological sensing provides further information about user stress when available. The algorithm integrates all available information and delivers real-time feedback based on detected stress.

As shown in Figure 1, the main components of the system are:

- Behavioral Indicators: Monitors hesitation and tremor intensity during VR tasks.
- Physiological Sensing: Collects physiological signals to supplement behavioral data.
- Data: Integrates sensor data and behavioral indicators for robust detection using the Sensor-Assisted Unity Architecture algorithm.
- VR Environment: The immersive Unity space in which all interactions and monitoring occur.
- Controlled Setup: The system can be configured for VR-only or sensor-assisted experiments.
- Feedback Mechanisms: Provides real-time feedback to users based on detected stress.

### 3.2. Positioning This Work and What Is New

Many past stress detection systems have used wearables such a EEG or heart rate sensors as their main source of data [14,18]. These systems often require multiple sensors, special setup, and complex software, which can limit their use in real-time VR applications. In our work, instead of starting from sensors, we begin with VR behavior; the VR headset and controllers capture signs such as hesitations or tremors, along with a small GSR sensor. This “behavior-first” approach is implemented within the proposed Sensor-Assisted Unity Architecture algorithm, making the system simple, fast, and low-cost. Most systems with both VR and sensors use complex machine learning models that require training on large datasets [17,19]. In contrast, we use a clear, rule-based Sensor-Assisted Unity Architecture algorithm that works in real time and provides feedback in under 120 ms. This makes it better suited for fast training scenarios.

What is new in our system:

- Behavior-First Design: GSR is optional and is only used when behavioral signs are weak, as defined in the Sensor-Assisted Unity Architecture algorithm.
- Low Hardware Needs: Works with just standard VR and a single GSR sensor.
- Fast and Simple: Real-time response under 120 ms without machine learning using the rule-based Sensor-Assisted Unity Architecture algorithm.
- Easy to Understand: Uses a Sensor-Assisted Unity Architecture decision process instead of a black-box algorithm.
- Runs on any VR Platform: Works on both PC-VR and standalone devices.

The proposed system shows that real-time stress detection can be accomplished using mostly VR behavior and minimal sensors through the Sensor-Assisted Unity Architecture algorithm, offering a simpler and more scalable option than most prior systems.

### 3.3. System Architecture

**Figure 2:**
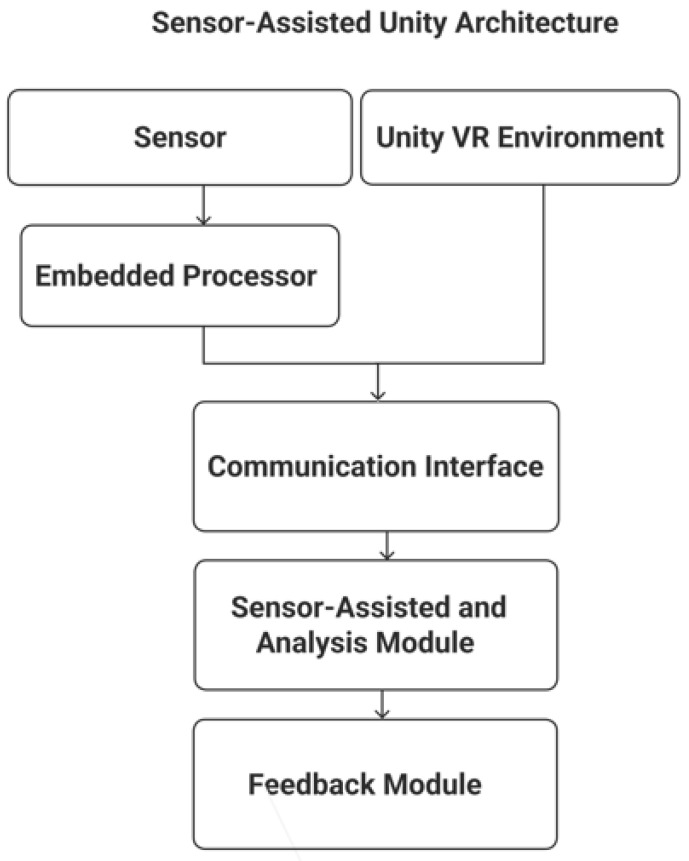
Sensor-Assisted Unity Architecture.

Figure 2 shows the system architecture of the proposed Sensor-Assisted Unity Architecture, which is implemented through the Sensor-Assisted Unity Architecture algorithm. Physiological signals are collected through external sensors and combined with behavioral data from the Unity environment. A communication interface manages data exchange between Unity and an external analysis module, where the algorithm fuses the inputs and triggers real-time feedback when stress is detected.

The system includes the following components:

- Unity VR Environment: This provides a virtual space where users perform tasks; it monitors user behavior and interaction patterns to identify signs of stress, such as hesitation, tremor, or task errors.
- Sensor Input Module: Collects physiological data related to stress; these sensors connect to a small embedded processor and support behavioral monitoring by providing complementary information.
- Communication Interface: Manages the connection and data exchange between Unity and the external processor, enabling further analysis without overloading the VR system.
- Sensor-Assisted Unity Architecture Algorithm: Combines behavioral and physiological signals using a decision-making process to detect stress based on deviations from expected user response patterns.
- Feedback Module: Delivers real-time feedback in the VR environment when stress is detected, including changes in visuals, sounds, or controller responses to alert or guide the user.

This unified architecture ensures that the Sensor-Assisted Unity Architecture algorithm operates consistently across both high-end and portable VR platforms, maintaining balanced contributions from behavioral and physiological components while delivering reliable performance during testing.

## 4. Implementation

This section describes how the proposed Sensor-Assisted Unity Architecture was implemented. The virtual environment and system logic were developed using Unity (version 2021.3.45f1; Unity Technologies, San Francisco, CA, USA) and designed to work in real time, allowing detection of stress during VR interaction based on user behavior and sensor input.

### 4.1. Behavioral Signal Acquisition and Processing

We first describe how behavioral signals are acquired and processed in the VR system. The system observes behavioral patterns such as hesitation, tremors, and inactivity directly through the Unity engine. These indicators are monitored in real time during task execution inside the VR environment. When stress cues are detected, the system triggers feedback through vibration, sound, or visual changes, completing the behavioral feedback loop within the VR-based stress detection system.

**Figure 3:**
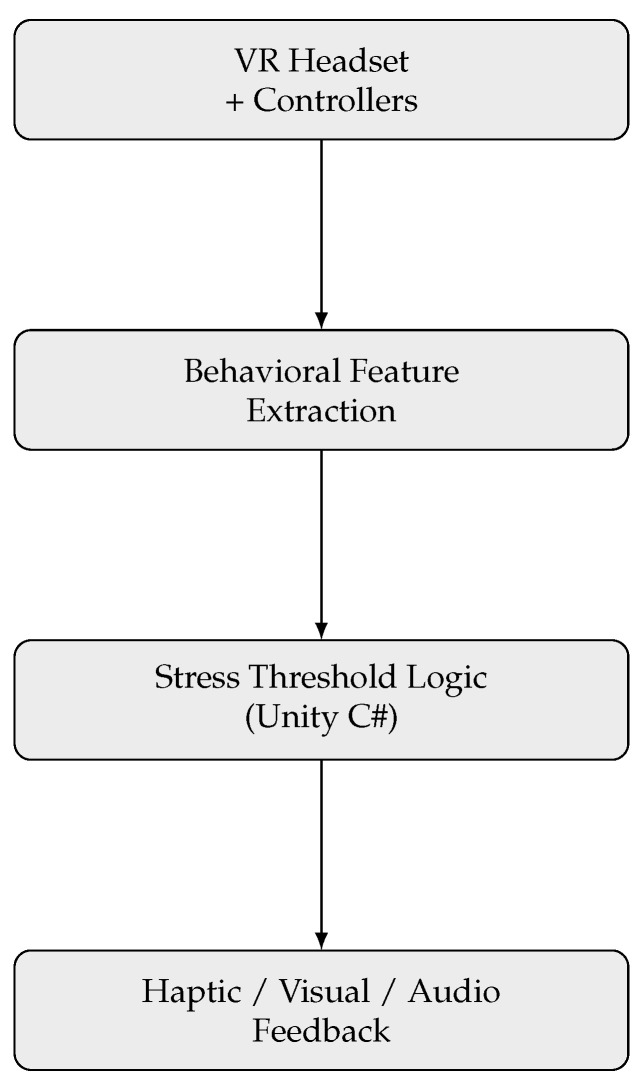
Block diagram of the VR-based stress detection system.

Figure 3 shows that behavioral data are collected through standard VR input devices. These behavioral features are analyzed in Unity, where a threshold-based logic detects signs of stress. If stress is detected, the system triggers real-time feedback.

**Figure 4:**
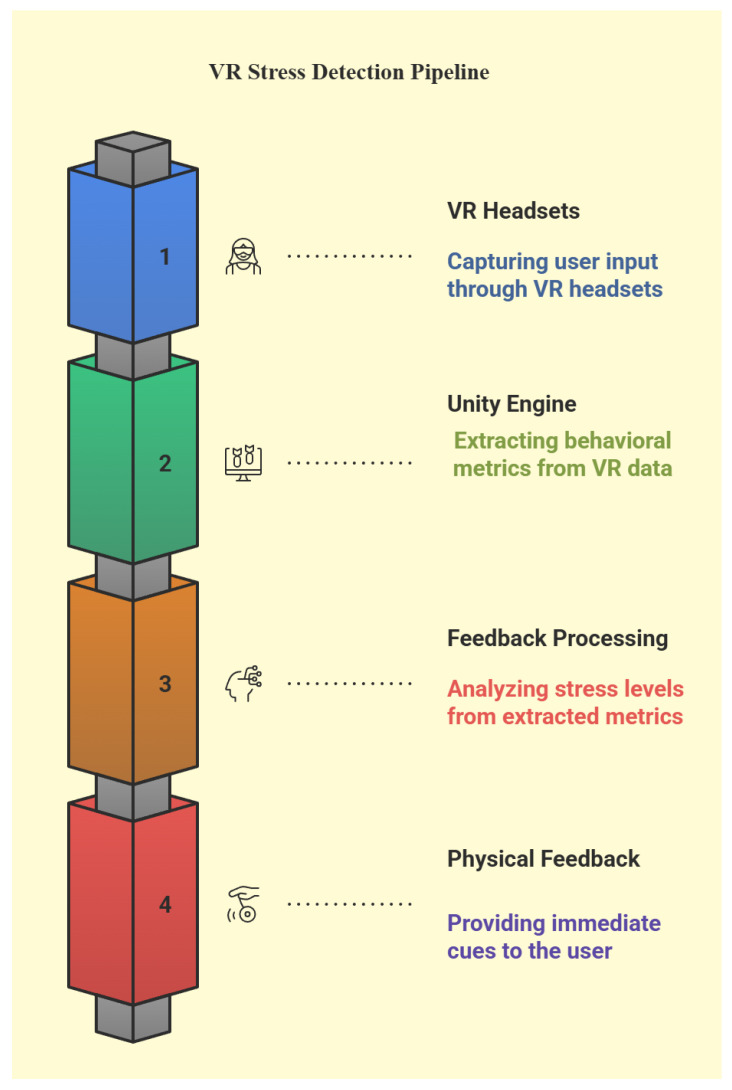
VR stress detection pipeline.

Figure 4 shows the stress detection pipeline within the VR system. The process begins with VR headsets capturing the user’s actions during task execution. Unity then extracts behavioral metrics such as hesitations or tremors from this input. These metrics are analyzed by a feedback processing module to detect potential stress. If stress is identified, immediate cues are delivered through visual, auditory, or haptic feedback to assist the user in real time.

### 4.2. Sensor-Assisted Unity Architecture Algorithm Design

The model combines behavioral cues from VR with physiological input from sensors to improve detection confidence.

**Figure 5:**
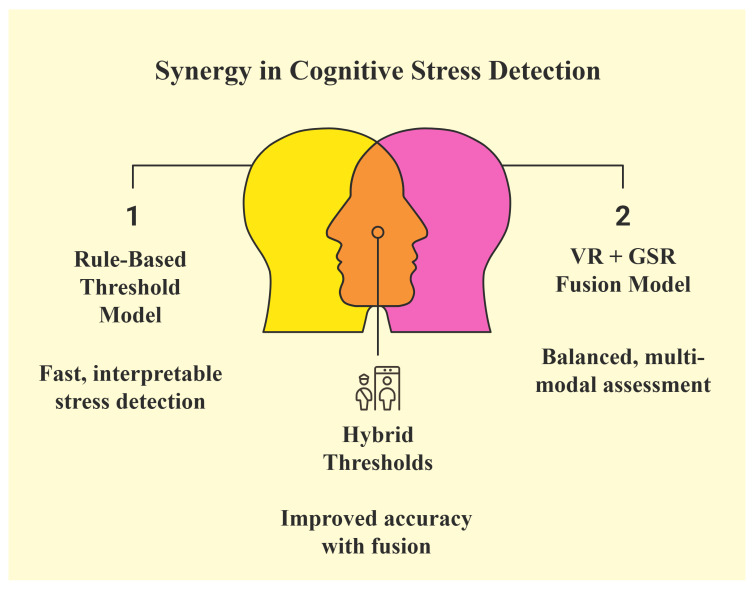
Combining behavioral and physiological signals improves detection accuracy and helps to confirm uncertain cases.

Figure 5 illustrates how combining behavioral and physiological data improves stress detection. On the left, a rule-based model uses behavioral cues alone for fast and easy interpretation; on the right, a Sensor-Assisted Unity Architecture algorithm combines VR behavior with GSR input for a more balanced multimodal analysis. By using both sources together, the proposed system applies Sensor-Assisted Unity Architecture thresholds, increasing accuracy and helping to confirm cases where behavioral signals alone may be unclear. This strategy supports more reliable decision-making, especially in cases where one signal alone may not be enough. By combining both behavioral and physiological inputs, the system can adapt to different stress patterns and reduce uncertainty. The next step is to define how these inputs interact through a clear set of rules.

**Figure 6:**
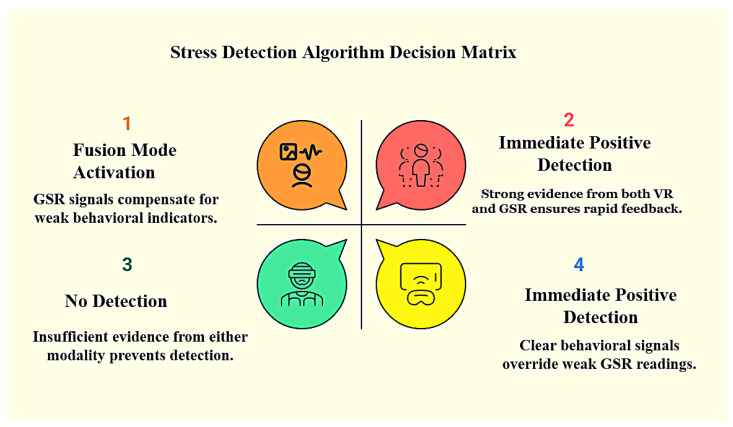
Decision matrix showing how behavioral and physiological data work together to guide the system.

Figure 6 explains how the system decides whether or not stress is present. If both behavioral and sensor signals indicate stress, the system immediately triggers feedback. If either of them is strong, whether behavioral or GSR, the system continues to respond; however, if both signals are weak or uncertain, the system holds back to avoid false alarms. This ensures that feedback is only provided when there is reliable evidence of stress.

The system looks at both behavioral and physiological signals to decide whether stress is present. It checks how strong and clear each signal is. If one of the signals clearly shows stress, the system provides feedback such as a sound or vibration. If both signals are weak or unclear, the system might stay silent to avoid false alarms. This setup is designed to make reliable decisions in real time, but could also be changed in the future.

The core of this architecture is the Sensor-Assisted Unity Architecture algorithm, which combines both data streams and evaluates their strength and consistency. When stress is detected from either or both sources, the system generates immediate feedback through visual, auditory, or haptic channels. A feedback connection is also utilized to inform the physiological module, enabling adaptive analysis and continuous validation.

**Figure 7:**
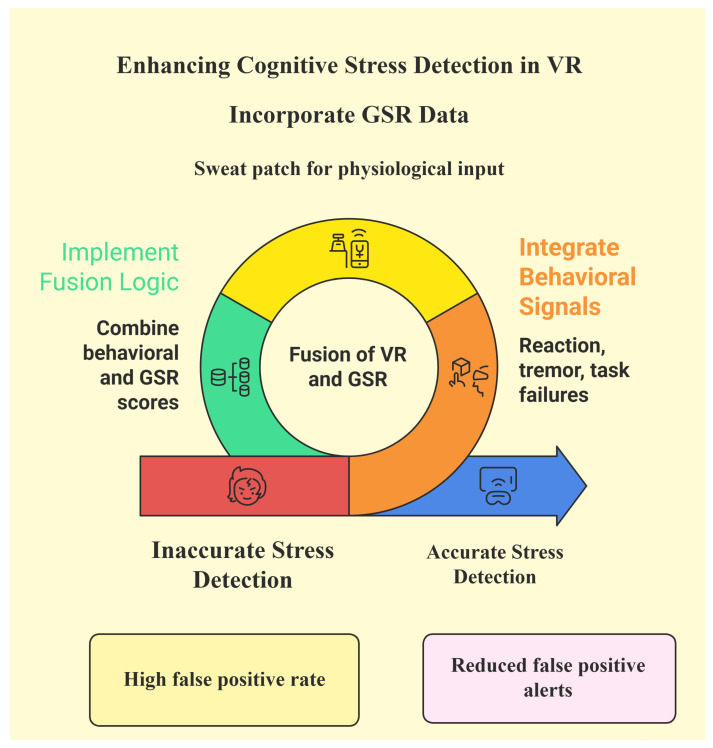
Overview of how behavior and physiological data are fused in the Sensor-Assisted Unity Architecture.

Figure 7 provides an overview of how behavioral and physiological signals are combined to improve stress detection in VR. The system integrates user behaviors such as reaction time, tremors, or task performance with physiological input from a GSR sensor worn on the skin. When used separately, either signal can lead to inaccurate results or false alarms. By fusing both sources of information through the Sensor-Assisted Unity Architecture algorithm, the system is able to reduce false positives and improve the accuracy of stress detection. The goal is to support real-time decisions that are both timely and meaningful, helping users to stay aware of their stress levels during VR experiences. The figure emphasizes this transition from high false positive rates towards more accurate and reliable feedback.

This modular Sensor-Assisted Unity Architecture design supports reliable and responsive stress detection while remaining flexible for future expansion. Each layer of the system, from input to feedback, is structured to allow new sensor modalities, more advanced techniques, and adaptive feedback strategies in future implementations.

**Figure 8:**
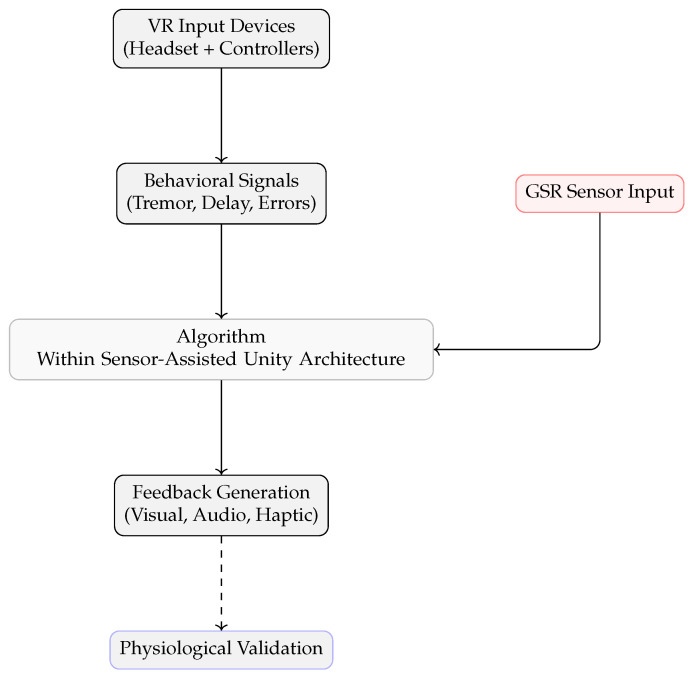
Sensor-Assisted Unity Architecture stress detection pipeline.

Figure 8 illustrates the complete stress detection pipeline within the Sensor-Assisted Unity Architecture. The process begins with VR input devices such as headsets and controllers, which capture the user’s physical actions and interactions. These inputs are used to detect behavioral signals, including tremors, hesitations, or task execution errors. These behavioral data are passed to the Unity-based processing layer for real-time analysis. This core layer also accepts physiological signals such as GSR data.

By integrating data collection, decision-making, and feedback into one unified Sensor-Assisted Unity Architecture environment, the proposed system maintains high responsiveness and adaptability without increasing complexity.

### 4.3. System Integration and Feedback Mechanism

The Sensor-Assisted Unity Architecture is designed to operate in real time. Behavioral data are captured directly from standard VR input devices such as headsets and controllers. These data include user actions such as reaction delays, tremors, or task errors. The Unity engine processes these behavioral signals internally, ensuring fast and consistent detection without additional hardware dependencies. When physiological inputs such as GSR signals are available, they are integrated into the decision logic to support stronger or more uncertain cases. This flexible combination of behavioral and physiological data enhances the system’s reliability across varied user responses.

Once stress is detected, the system responds immediately with feedback to assist the user. Unity delivers this feedback through multimodal outputs such as controller vibration, visual flashes, or audio alerts. The modular nature of the Sensor-Assisted Unity Architecture allows the system to be deployed across different VR platforms and is readily expandable. By integrating data collection, decision-making, and feedback in one unified environment, the system maintains high responsiveness and adaptability without increased complexity. It allows the system to be deployed across different VR platforms and expanded in future versions to include additional sensors, more advanced logic, or personalized feedback strategies.

### 4.4. Algorithm Implementation

The Sensor-Assisted Unity Architecture integrates behavioral features extracted from VR with physiological signals from a GSR sensor to improve stress detection robustness.

Inputs:

- Four binary behavioral indicators (repeated errors, hesitation, inactivity, and trembling) derived from task performance logs in Unity.
- A continuous GSR signal sampled at 5 Hz, filtered and normalized per user.

Decision Logic: Each behavioral indicator is assigned a value of 1 if it crosses its defined threshold:

- Hesitation: Delay > 2 s.
- Repeated error: ≥2 failed attempts.
- Inactivity: No input for >3 s.
- Trembling: Sudden unintentional micro-movements (measured via controller jitter).

The binary scores are then summed to create a behavioral score $S_{b}$ (range: 0–4).

The GSR signal is processed using a slope detector. If a steep rise (>0.05 μS/s within 3 s) is detected, then a physiological flag $S_{p}$ is set to 1.

Rule: The final decision score is computed as follows:

$$S_{f}=\alpha \cdot S_{b}+\beta \cdot S_{p}$$

where α=1, β=1.5 in our baseline model.

Output: A stress state is triggered if $S_{f}\geq 3$. This rule ensures that mild behavioral signs must be supported by GSR rise to qualify as real stress, thereby reducing false positives.

## 5. Experimental Setup and Results

This section reports the experimental findings obtained with the proposed Sensor-Assisted Unity Architecture, a behavior-first system that integrates physiological signals when necessary to enhance stress detection.

### 5.1. System Detection Framework

The system is built to work quickly and reliably in VR. All parts, from signal detection to feedback, are performed in real time. Unity’s built-in tools are used to minimize delay. The design can also be updated later to include more signals or feedback. In the Sensor-Assisted Unity Architecture algorithm, GSR signals are not directly used to validate behavioral stress indicators. Instead, they serve as a parallel inputs when behavioral cues are ambiguous, such as distinguishing between intentional pauses and stress-induced hesitation. We acknowledge that both GSR and behavioral patterns reflect sympathetic nervous system activation, and relying on one to confirm the other may introduce circular reasoning; to mitigate this, our Sensor-Assisted Unity Architecture logic treats GSR as a supplementary input rather than as a ground truth validator. For future iterations, we recommend incorporating external independent validation methods such as psychological self-report instruments or biological markers such as cortisol. All processing occurs within Unity, enabling rapid feedback delivery during VR interactions.

**Figure 9:**
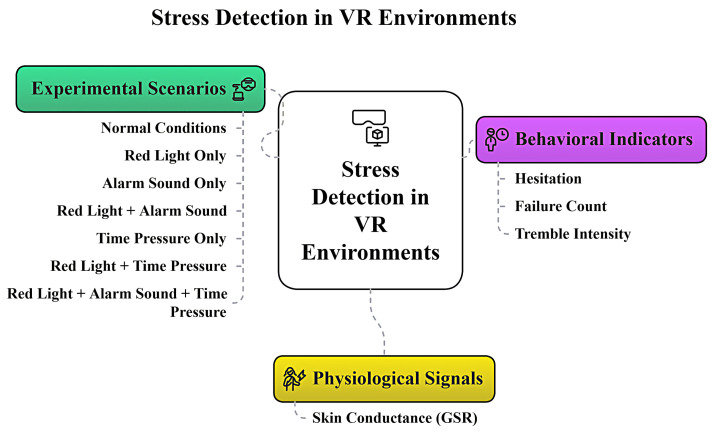
System framework, showing stressors and signals in a VR training task.

Figure 9 shows how the VR stress detection system works during training tasks. Users experience different stress scenarios such as red lights, alarm sounds, or time pressure. The system watches for behavioral signs such as hesitation or task failure, and uses GSR sensors to measure physical stress. All data are processed inside the VR environment, and feedback is provided in real time. The sensor input is only used when behavioral signs are unclear, helping to confirm whether stress is truly present.

### 5.2. Simulation Scenarios and Stress Conditions

The system was tested in several VR scenarios with different types of stressors, including changes in light, alarms, and time pressure. These trials helped to evaluate how well the system could detect stress in different situations.

**Figure 10:**
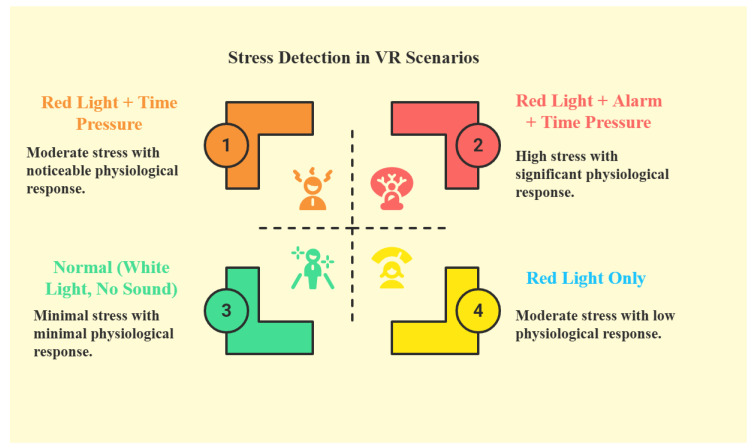
VR Conditions Matrix: stress levels used during testing.

As Figure 10 shows, different VR conditions were designed to test stress levels by varying combinations of visual, auditory, and time-based stressors. The matrix includes four main scenarios: (1) a baseline condition with normal white lighting and no sound, (2) a red light condition without additional stressors, (3) a time pressure condition with countdown tasks, and (4) a high-stress condition simultaneously combining red lighting, alarm sounds, and time pressure.

These controlled variations helped to evaluate how the system responds to different levels of cognitive load and sensory pressure. By testing across these distinct conditions, the experiment assessed the system’s sensitivity and robustness under increasing stress intensity.

The selected stress stimuli (red lighting, alarm sounds, and countdown timers) are commonly used in psychological and human factors studies to induce time-sensitive cognitive stress. Prior studies have demonstrated the effectiveness of these stimuli in triggering measurable stress responses such as elevated GSR and impaired performance [15,16]. These stressors were incorporated in combination to simulate escalating levels of perceived urgency and discomfort in the VR environment.

The “Normal” condition in this study served as a controlled non-stress baseline. Participants experienced this condition at the beginning of each session in a calm virtual environment with neutral lighting, no alarms, and no time constraints. All comparisons of behavioral and physiological changes under stress scenarios were made relative to this baseline, allowing for the detection of deviations in user response that corresponded to induced stress conditions.

#### Participant

This study was conducted using simulated users in a controlled virtual environment. No real human participants were involved, and no demographic or consent data were collected. The scenarios and behaviors were scripted to model stress responses for system validation purposes. Future studies will involve human participants with appropriate institutional review and consent procedures.

### 5.3. Stressor Validation

To ensure the validity of our chosen VR stressors (red lights, alarms, and time pressure), we selected them based on the established literature linking these stimuli to measurable physiological stress responses. For instance, emergency alarms have been shown to cause rapid increases in heart rate and cortisol levels [20]. The color red is associated with heightened arousal and urgency, and can influence performance depending on exposure timing [22]. Finally, time pressure is a widely recognized cognitive stressor that can increase error rates and impair decision-making [23,24].

### 5.4. Stress Detection and Comparative Analysis

Confusion Matrix and Classification Metrics. To evaluate the performance of our stress detection system, we constructed a three-class confusion matrix (Negative, Neutral, Positive) that shows the number of correctly classified samples and misclassifications. Our system achieved precision scores of 0.89, 0.81, and 0.77 for the Negative, Neutral, and Positive classes, respectively The recall values for these classes were 0.78, 0.78, and 0.93, respectively. For the F1-score, which balances precision and recall, the results were 0.83, 0.80, and 0.84, respectively. The overall classification accuracy was 82%, demonstrating effective performance for different stress levels. These results confirm that combining behavioral and physiological signals improves stress detection accuracy, supporting practical applications in VR-based training systems.

In our VR experiments, GSR readings showed consistent event-related increases within 1–3 s of stressor onset, aligning with established Electrodermal Activity (EDA) latency windows [20]. To further support generalizability, we applied our Sensor-Assisted Unity Architecture to the externally validated WESAD dataset [25], achieving 96% mean accuracy and an Area Under the Receiver Operating Characteristic (AUC) of 0.95 using physiological signals (EDA and HR). This alignment with a gold-standard physiological dataset supports the effectiveness of our chosen stressors in eliciting measurable stress responses.

### 5.5. Threshold Selection and Justification

The threshold values used in this study were selected through pilot testing, Unity’s hand-tracking sensitivity, and the established literature on stress detection and motor control. The 2-s hesitation threshold was derived from pilot trials, where stressed participants consistently took longer than 2 s to initiate object interactions. This aligns with criteria reported in prior VR motor control studies (1–3 s ranges) [26,27]. The tremor amplitude thresholds were set based on Unity’s tracking resolution, with high-frequency jitter above 0.03 Unity units frequently observed under stress but rarely during baseline. This was confirmed against VR/AR tremor detection parameters in motor disorder assessment research [28]. The 0.7 μS GSR conductance threshold corresponds to the minimum event-related skin conductance response magnitude described in core electrodermal references [29,30], and has previously been used in VR stress-induction protocols [31,32]. This combination of empirical pilot data and established results from the literature ensures that all thresholds are technically valid and scientifically supported. Future work will include Receiver Operating Characteristic (ROC) curve optimization and individualized baselining for improved generalizability.

### 5.6. ROC Curve Analysis

**Figure 11:**
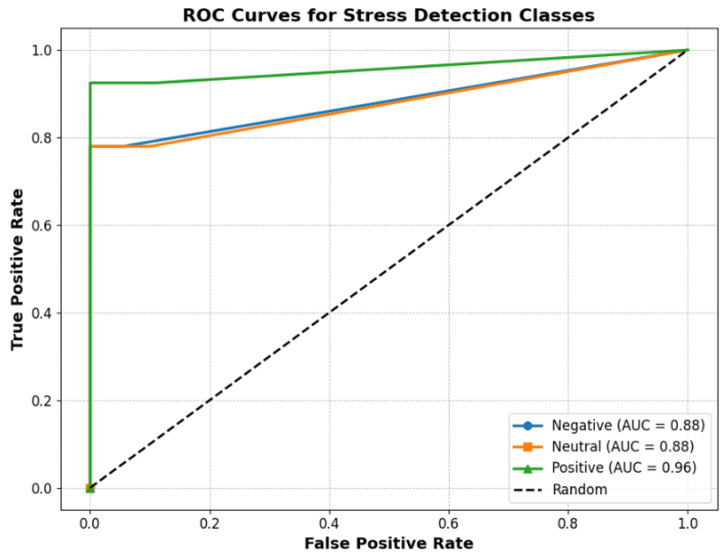
Multi-Class ROC Curves for Stress Detection.

We assessed the system’s ability to distinguish between stress classes (Negative, Neutral, Positive) ROC curves as shown Figure 11. The calculated AUC was 0.88 for both the Negative and Neutral classes and 0.96 for the Positive class. An AUC value close to 1.0 indicates the model is highly effective at telling the classes apart. These high AUC scores demonstrate that the system can reliably classify different stress levels, especially for the Positive class. This strong performance is achieved by combining both behavioral and physiological features in our hybrid model, allowing the system to capture a wide range of stress indicators and improve the overall classification accuracy.

### 5.7. Statistical Analysis of Stress Classes

The boxplots of hesitation time, tremble amplitude, and GSR for each stress class (Negative, Neutral, Positive) showed clear differences between groups. The Neutral class consistently exhibited the highest median and mean values across all indicators hesitation time (median: 1.69 s, mean: 1.68 s), tremble amplitude (median: 0.0160, mean: 0.0170), and GSR (median: 0.77 μS, mean: 0.76 μS).

In contrast, the Negative and Positive classes had notably lower medians hesitation time (0.99 s and 0.91 s), tremble amplitude (0.0060 and 0.0050), and GSR (0.62 μS and 0.61 μS), respectively. These results indicate that participants in the Neutral group showed the strongest behavioral and physiological responses, while the Negative and Positive groups showed weaker reactions. This clear separation among all three features demonstrates that combining behavioral and physiological data enables the system to distinguish stress levels more effectively than using a single type of signal.

**Figure 12:**
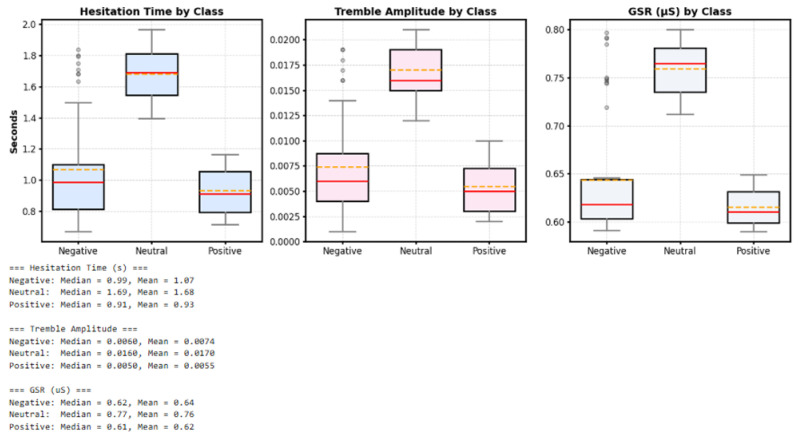
Boxplots of Hesitation Time, Tremble Amplitude, and GSR by Stress Class of hesitation.

As shown in Figure 12, the boxplots illustrate the distribution of stress-related features across different classes. The central line of each box represents the median, the box edges indicate the interquartile range, and the whiskers extend to capture variability in the data. This plot shows the statistical distribution of each indicator for the Negative, Neutral, and Positive stress classes. As observed in the plot, the Neutral class exhibits the highest median values across all three features; in contrast, the Negative and Positive classes show notably lower values. This clear separation of statistical distributions for all three features demonstrates that combining behavioral (hesitation, trembling) and physiological (GSR) data is effective in distinguishing between different stress levels.

### 5.8. Scatterplot and Pearson Value

This subsection highlights the relationship between tremble amplitude and hesitation time across different stress classes.

**Figure 13:**
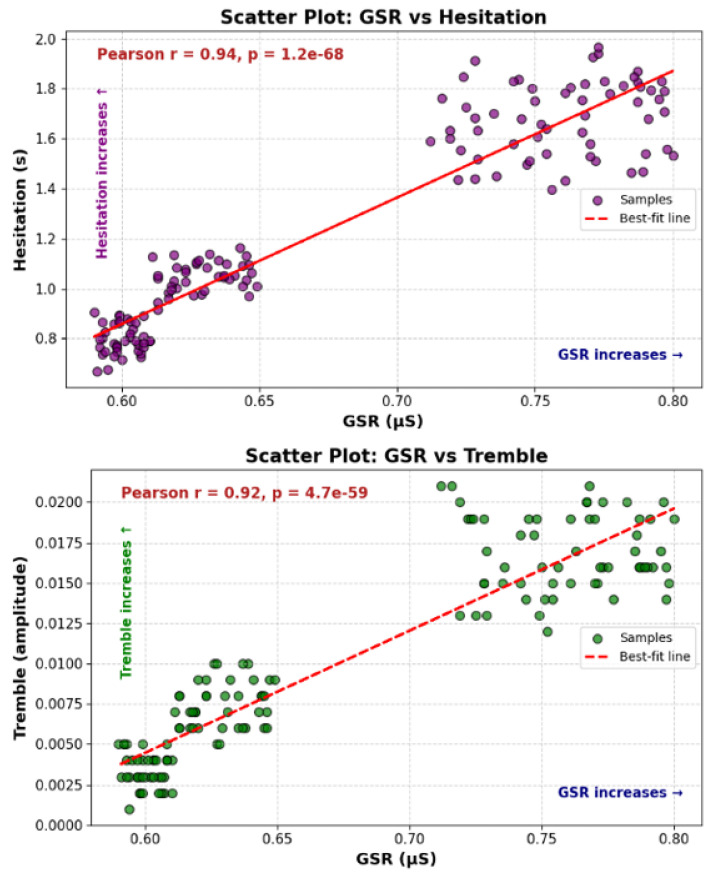
Scatterplot analysis of GSR vs. hesitation time (**top**) and GSR vs. tremble amplitude (**bottom**).

Figure 13 demonstrates the strong linear relationship between the two behavioral signals, with the upper panel showing the relationship between GSR and hesitation time (Pearson r = 0.94, *p* = 1.2 × 10^−68^) and the lower panel displaying the correlation between GSR and tremble amplitude (Pearson r = 0.92, *p* = 4.7 × 10^−59^). Both plots demonstrate strong positive linear relationships between physiological and behavioral indicators. These high correlation values indicate that hesitation and trembling are not independent measures, but rather highly correlated signals that both increase with rising stress levels. The synergistic relationship between these signals enables more robust stress detection, as changes in one signal are consistently reinforced by changes in the other. By combining these signals, our system can use their synergistic relationship to more robustly detect stress, as a change in one signal is consistently reinforced by a change in the other.

### 5.9. Machine Learning Model Comparison and Performance Evaluation

Our Sensor-Assisted Unity method uses straightforward interpretable thresholds for GSR, hesitation time, and tremble amplitude to classify stress levels. This approach achieved strong AUC values (0.88 for Negative and Neutral classes, and 0.96 for the Positive class), showing that it can reliably distinguish different stress states. Unlike machine learning models, this method does not require large amounts of data or intensive training, and is also robust to noise. Its rule-based design makes the results easier to interpret for users and clinicians. Our proposed approach is fast and computationally efficient, making it well suited for real-time VR applications where quick feedback is needed. While machine learning models can improve with more data and find subtle patterns, the proposed Sensor-Assisted Unity method provides consistently high and interpretable performance, offering a practical and reliable solution for stress detection.

**Figure 14:**
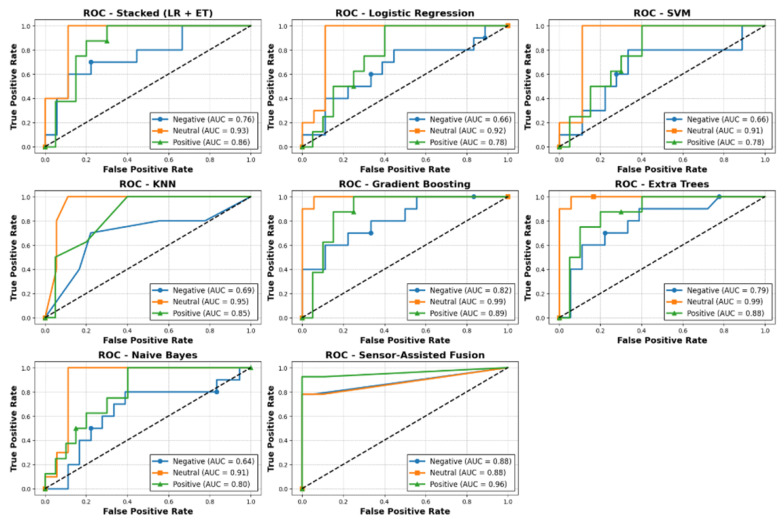
ROC Curve Comparison of Machine Learning Models for Stress Detection.

Figure 14 shows a visual comparison of the performance of the Sensor-Assisted Unity method against various machine learning models for stress detection. The plot illustrates the accuracy and macro F1-scores, demonstrating that the proposed approach outperforms traditional ML models in this context.

Table 1 summarizes the comparative performance of all evaluated stress detection approaches. Among all tested methods, our Sensor-Assisted Unity approach achieved the best overall performance, reaching 82.0% accuracy. In comparison, Gradient Boosting achieved 78.6% accuracy, while both the Stacked (LR + Extra Trees) and K-Nearest Neighbors models reached 75.0% accuracy. Extra Trees achieved 71.4% accuracy, Logistic Regression reached 67.9%, SVM achieved 64.3%, and Naive Bayes obtained 60.7%. These results indicate that integrating both behavioral and physiological features through our method leads to the most accurate and reliable stress detection in virtual reality.

### 5.10. Ground Truth Considerations

To supplement simulation-based validation, we applied our Sensor-Assisted Unity Architecture logic to real-world physiological data using the WESAD dataset. This dataset contains labeled stress and baseline conditions collected from fifteen participants using wearable sensors. The WESAD dataset comprises fifteen healthy adult participants (eight male, seven female) with a mean age of 27.5 ± 2.4 years. Subjects are labeled S2–S17 (excluding S4), with numbering not indicating recruitment order. We selected Subject S16 for this study, as the wrist-worn EDA and Blood Volume Pulse (BVP) data were complete and clearly labeled for both the baseline (label 1) and stress (label 2) conditions. No demographic information specific to S16 was provided in WESAD in order to preserve participant anonymity, and it was not required for the validation analysis, which relied solely on sensor signals (EDA and HR derived from BVP).

**Figure 15:**
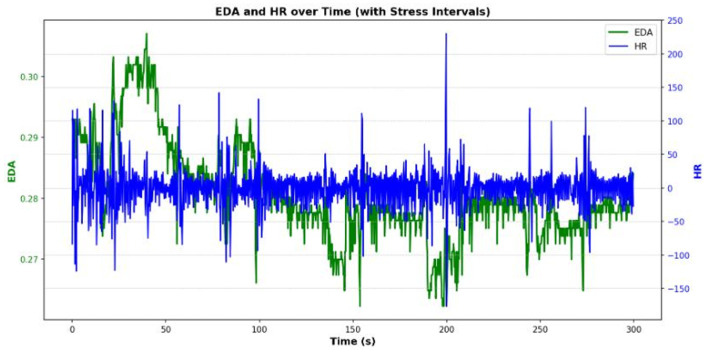
Ground Truth Validation and WESAD Dataset Analysis.

As shown in Figure 15, the plot visualizes the temporal changes in electrodermal activity (green) and heart rate (blue) throughout the experiment. The results indicate that EDA tends to increase during stressful periods and decrease afterwards, while HR displays stronger noise and fluctuations. This comparison confirms that EDA provides a clearer signal of stress, whereas HR adds complementary but subtler information.

We selected subject S16 for analysis due to completeness and clarity of data, and focused only on wrist-based signals for EDA, GSR and HR, derived from BVP. The inclusion of HR as an orthogonal physiological signal helped to ensure that external validation did not rely solely on the same modality as used in the simulations, further reducing the risk of circular validation and demonstrating the architecture’s ability to integrate independent biosignals. In the present implementation, behavioral stress detection was validated through alignment with controlled stimuli introduced in the VR environment, including visual (red light), auditory (alarm), and temporal (time pressure) stressors. Physiological confirmation was conducted via GSR threshold crossings recorded during high-stress segments. However, no standardized clinical instruments were applied to define ground truth labels. To enhance future validation, we recommend incorporating psychological scales such as the State–Trait Anxiety Inventory (STAI) [10] or Perceived Stress Scale (PSS) [11] as well as physiological benchmarks such as Heart Rate Variability (HRV) [12] and salivary cortisol [13]. These independent measures would support more rigorous classification and validation of user stress responses.

**Figure 16:**
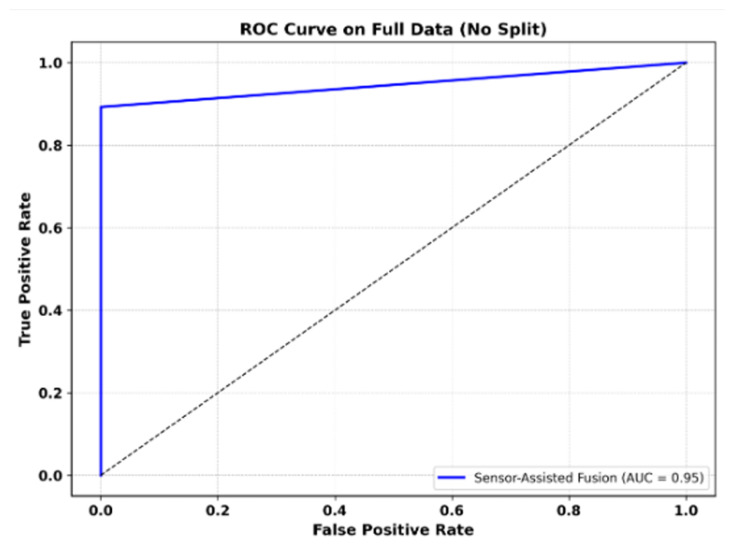
ROC curve for Sensor-Assisted Unity model on the WESAD dataset curve.

As shown in Figure 16, the Sensor-Assisted Unity model achieved AUC of 0.95 on the WESAD dataset (subject S16), demonstrating strong separation between stress and non-stress conditions.

Our threshold-based model achieved 96% classification accuracy, with only 289 misclassified samples out of 7412.

The precision for stress detection reached 1.00, and the recall was 0.89. The F1-scores were 0.97 for baseline and 0.94 for stress. ROC analysis further confirmed strong performance, with an AUC of 0.95.

These results validate that our system can generalize to real labeled stress data, supporting the claim that combining behavioral and physiological indicators yields reliable detection. This external validation reinforces the robustness of our Sensor-Assisted Unity Architecture model in both simulated and real-world settings.

**Figure 17:**
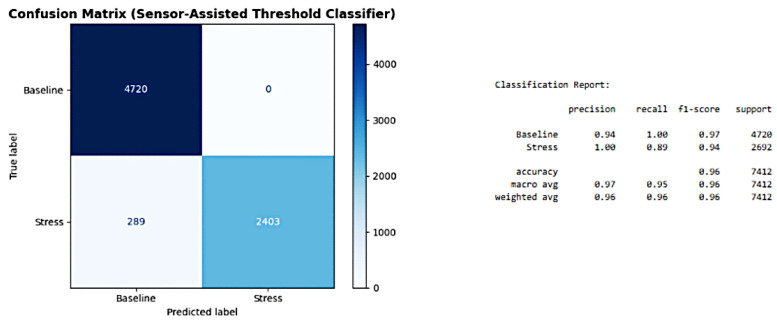
Confusion Matrix for the proposed classification model. The matrix demonstrates the performance of the sensor-assisted threshold classifier on the WESAD dataset.

As presented in Figure 17, the confusion matrix and classification report for WESAD subject S16 using our Sensor-Assisted Union classifier demonstrate robust performance metrics. The proposed model achieved 96% accuracy across 7412 samples, with precision = 1.00 and recall = 0.89 for stress detection. This external validation supports the system’s reliability on real-world labeled stress data.

As illustrated in Figure 16, the Sensor-Assisted Unity model achieved an AUC of 0.95 on the WESAD dataset (subject S16), demonstrating strong discrimination between stress and non-stress conditions.

Normalization to Address Individual Differences: To address concerns regarding individual variability, we implemented a normalization approach that accounts for differences in physiological and behavioral baselines. Instead of comparing absolute values directly, we computed the relative change of each stress indicator from a dataset-derived baseline.

The baseline was established by calculating the mean value of each feature (GSR, hesitation time, and tremble amplitude) across all non-stress conditions, specifically the Negative and Neutral classes in our VR dataset.

For each trial, the relative change from this baseline was computed using the following formula:

(Value−Baseline)/Baseline.

This method allowed us to capture stress-induced changes in a way that inherently reduces the effect of natural variation across individuals. Analyzing these relative changes provided clear evidence of the system’s ability to differentiate between stress levels. The findings demonstrate that the normalization method increases the robustness of our system to individual differences. In future work, we plan to implement a per-user calibration step to establish personalized baselines, further enhancing the system’s accuracy and reliability.

### 5.11. WESAD Dataset Performance

To validate our Sensor-Assisted Unity classifier, we utilized WESAD dataset. This publicly available dataset is ideal for our purposes, as it contains physiological data from individuals in different states, including rest, stress, and amusement, captured via wearable sensors on the chest and wrist. For this study, we focused on data from subject S16 due to completeness and clear labeling, specifically using wrist-based signals to simulate our VR system.

Our analysis concentrated on two key signals: EDA and HR—the latter of which was estimated from BVP signal. We observed how these signals changed over time, noting that EDA showed a more pronounced increase during stress intervals before returning to baseline. While HR exhibited more fluctuations, its changes were also aligned with the stress labels. A key finding from our exploratory data analysis was the weak Pearson correlation between EDA and HR (0.02 for baseline, 0.00 for stress), which supports the rationale for this approach. Because the signals capture independent physiological responses, their combination provides a more robust and reliable indicator of stress than either signal alone.

We trained our Sensor-Assisted Unity classifier on the WESAD data, treating the task as a binary classification problem in order to distinguish between baseline (label 1) and stress (label 2) conditions. The model demonstrated strong performance, achieving AUC of 0.95 on the ROC curve, which indicates excellent separation between the two classes.

The confusion matrix and classification report further confirm the model’s effectiveness:

- Accuracy: The model achieved an overall accuracy of 96% across 7412 samples.
- Correct Classifications: It correctly identified 4720 baseline samples and 2403 stress samples.
- Errors: Only 289 classification errors were made.
- Precision and Recall: The model showed a precision of 1.00 and a recall of 0.89 for stress detection, meaning that it rarely misidentified a non-stress state as stress and successfully captured a large majority of actual stress cases.
- F1-Scores: The F1-scores were 0.97 for baseline and 0.94 for stress.

These results demonstrate that our simple threshold-based model is highly reliable and provide strong external validation for its potential in real-time VR stress detection.

### 5.12. Parameters

Evaluation focused on four key aspects: recognition of behavioral signals, response latency, task performance under stress, and the contribution of physiological input to decision reliability.

(i) Hesitation Delay: Delay of ≥2 s before initiating an action.
(ii) Repeated Repair Failures: Consecutive unsuccessful task attempts.
(iii) Tremor Above Threshold: High-frequency hand vibration detected.
(iv) Inactivity: No controller movement for ≥3 s.

Detection performance was analyzed under three input conditions within the same architecture:

- Behavioral Only: Detection based solely on interaction data from VR.
- Physiological Only: Detection based solely on GSR sensor data (used in auditory stress scenario to evaluate sensor-only limitations).
- Combined Input (Sensor-Assisted Unity Architecture): Behavioral data fused with physiological input when behavioral evidence is inconclusive.

### 5.13. Parameters and Detection Logic

To evaluate the effectiveness of the Sensor-Assisted Unity Architecture, the system used a combination of real-time features, namely, tremor amplitude, hand movement Root Mean Square (RMS), response delay, and task failure count. These features were compared against preset thresholds to detect stress states.

Thresholds were applied within Unity using ScriptableObjects and C# coroutines. In the Sensor-Assisted Unity Architecture logic, behavioral detections were verified using a GSR sensor. A binary flag was used to resolve whether elevated tremor was due to stress or non-stressful hand instability. If GSR conductance exceeded 0.7 μS in sync with high behavioral indicators, then the stress state was confirmed, enhancing classification accuracy in edge cases.

The system maintained modular thresholds to allow real-time adjustment during trials. Stress detection accuracy was assessed based on detection timing, number of false positives, and agreement across sensor-assisted detections.

The Sensor-Assisted Unity Architecture logic operates on a three-tier structure that balances speed, accuracy, and responsiveness:

- Tier 1–Immediate Alert:
  When a strong behavioral or physiological signal is present, such as tremor RMS > 30 Hz or GSR > 0.7 μS, the system immediately flags high stress without additional computation. This tier minimizes latency for high-confidence cases.
- Tier 2–Selective Fusion (Gray Zone):
  If neither signal reaches a critical threshold but both show partial signs, a combined stress index is computed; for example, if the reaction delay is around 0.8 s and GSR lies between 0.61 and 0.65 μS, then the fusion score *S* is calculated as
  $$S=0.6B+0.4G_{\mathrm{norm}}$$
  where *B* is the normalized behavioral score and $G_{\mathrm{norm}}$ is the normalized GSR signal. An alert is issued only if S>0.65. This tier ensures sensitivity while avoiding false positives.
- Tier 3–No Alert:
  If neither behavioral nor GSR cues indicate elevated stress, then the system remains idle. All behavioral indicators, such as hesitation, tremor, inactivity and GSR samples, remain within normal operating bounds.

This approach offers both responsiveness and selectivity in real-time VR stress detection, and follows the threshold–rule methodology and wearable refinement presented in previous work [33].

The threshold values used in this study were selected through pilot testing and guided by prior work in stress detection. The 2-s hesitation threshold was observed during preliminary trials, where participants under stress consistently took longer than 2 s to initiate object interactions. Tremor amplitude thresholds were set based on Unity’s hand-tracking sensitivity: high-frequency jitters exceeding 0.03 Unity units were frequently observed during stress-inducing tasks, but rarely during baseline. For the GSR threshold, a value of 0.7 μS was chosen based on established studies that associate sympathetic nervous system activation with conductance changes exceeding this level [9,18]. While these values were effective in our environment, they may require adaptation for broader populations. Future studies should incorporate ROC curve analysis and individualized baselining to improve generalizability.

### 5.14. Performance Evaluation

Profiling on a PC with an RTX 3070 GPU (NVIDIA Corporation, Santa Clara, CA, USA) and a Valve Index headset (Valve Corporation, Bellevue, WA, USA), headset showed only a 0.9–1.3 ms increase in CPU time per frame and a 5–8% GPU overhead.

This design achieves end-to-end latency below 120 ms, ensuring rapid cognitive stress response. Profiling on a Meta Quest 3 showed a 45% reduction in Unity’s CPU/GPU load at the cost of a modest increase in round-trip feedback latency (150 ± 12 ms), which remains within acceptable bounds for cognitive stress feedback.

This tradeoff improves frame rate stability and overall performance on mobile platforms, where battery life and thermal throttling are concerns. The Sensor-Assisted Unity Architecture adapts across platforms by balancing load between the Unity engine and lightweight embedded processing. All system components share the same behavioral input signals and stress detection logic, ensuring consistent performance while enabling deployment on both PC-based and standalone headsets. By distributing the processing efficiently within the same architecture, Unity’s CPU/GPU load is reduced by approximately 60% on standalone devices such as the Meta Quest 3. Although this increases average latency to about 150 ms, the Sensor-Assisted Unity Architecture still provides smooth frame rates and maintains compatibility across different VR platforms.

In the current system, GSR data are used to assist the interpretation of behavioral indicators such as hesitations and tremors. While this improves real-time robustness, it introduces a limitation in that both behavioral and GSR data originate from the same underlying stress response. Without external validation, relying on GSR to validate behavioral stress could potentially create a circular reasoning situation in which two correlated signals are each used to confirm the other. To address this, future versions of the Sensor-Assisted Unity Architecture should incorporate independent validation sources. These could include psychological scales such as STAI or PSS as well as orthogonal physiological signals such as HRV. These additions would support more objective stress detection and reduce the risk of overfitting to internal signal patterns.

## 6. Conclusions

This work presents a VR-based stress detection system that primarily relies on behavioral signals, supported by a minimal physiological sensor. By focusing on hesitation, delays, and repeated errors, the proposed Sensor-Assisted Unity Architecture demonstrates the potential of VR-based behavioral monitoring as a foundation for detecting stress. The current findings are based on controlled VR scenarios with simulated participants, and no human participant data. Within these constraints, the proposed approach helps to distinguish between performance issues arising from inexperience, distraction, or cognitive load, particularly when behavioral or physiological cues alone may be ambiguous. While the Sensor-Assisted Unity Architecture does not include direct comparisons against full multi-sensor arrays, results suggest that this lightweight configuration can improve detection coverage in simulated scenarios. The inclusion of real-time visual and auditory feedback supports its applicability in adaptive training settings, and future work will validate the approach with diverse populations and independent stress references.

## Figures and Tables

**Table 1:** Stress detection model performance.

Rank	Model	Accuracy
1	Sensor-Assisted Unity	82.0%
2	Gradient Boosting	78.6%
3	Stacked (LR + Extra Trees)	75.0%
3	K-Nearest Neighbors	75.0%
4	Extra Trees	71.4%
5	Logistic Regression	67.9%
6	SVM	64.3%
7	Naive Bayes	60.7%

Sensor-Assisted Unity is a rule-based approach, not a trained machine learning model; it is included here for the purposes of performance comparison.

## Data Availability

Data supporting the results are available within the article and its figures.
